# Fibrosis in Chronic Kidney Disease: Pathogenesis and Consequences

**DOI:** 10.3390/ijms22010408

**Published:** 2021-01-02

**Authors:** Sara Panizo, Laura Martínez-Arias, Cristina Alonso-Montes, Pablo Cannata, Beatriz Martín-Carro, José L. Fernández-Martín, Manuel Naves-Díaz, Natalia Carrillo-López, Jorge B. Cannata-Andía

**Affiliations:** 1 Bone and Mineral Research Unit, Hospital Universitario Central de Asturias, Instituto de Investigación Sanitaria del Principado de Asturias (ISPA), Retic REDinREN-ISCIII, Universidad de Oviedo, 33011 Oviedo, Spain; sarapanizogarcia@gmail.com (S.P.); lauramartinezarias@gmail.com (L.M.-A.); cristinaam.huca@gmail.com (C.A.-M.); bea_m15@hotmail.com (B.M.-C.); jlfernandez.huca@gmail.com (J.L.F.-M.); ncarrillolopez.huca@gmail.com (N.C.-L.); 2 Pathology Department, Fundación Instituto de Investigaciones Sanitarias-Fundación Jiménez Díaz (IIS-FJD), Universidad Autónoma de Madrid (UAM), Retic REDinREN-ISCIII, 28040 Madrid, Spain; pablocannata@gmail.com

**Keywords:** fibrosis, inflammation, RAS, PTH, FGF23, Klotho, microRNAs, vitamin D, artificial intelligence, image analysis

## Abstract

Fibrosis is a process characterized by an excessive accumulation of the extracellular matrix as a response to different types of tissue injuries, which leads to organ dysfunction. The process can be initiated by multiple and different stimuli and pathogenic factors which trigger the cascade of reparation converging in molecular signals responsible of initiating and driving fibrosis. Though fibrosis can play a defensive role, in several circumstances at a certain stage, it can progressively become an uncontrolled irreversible and self-maintained process, named pathological fibrosis. Several systems, molecules and responses involved in the pathogenesis of the pathological fibrosis of chronic kidney disease (CKD) will be discussed in this review, putting special attention on inflammation, renin-angiotensin system (RAS), parathyroid hormone (PTH), fibroblast growth factor 23 (FGF23), Klotho, microRNAs (miRs), and the vitamin D hormonal system. All of them are key factors of the core and regulatory pathways which drive fibrosis, having a great negative kidney and cardiac impact in CKD.

## 1. Fibrosis and Chronic Kidney Disease (CKD)

Fibrosis is considered as an excessive accumulation of matrix connective tissue components. It can affect several organs like the skin, eye lungs, kidney, pancreas, liver, and heart [1]. Fibrosis is the final pathological process of a maladaptive repair, defined by the formation and accumulation of the extracellular matrix, mainly local mesenchymal cells. It is a process which is a response to different types of tissue injury, essential for the normal healing, closely associated with inflammation and tissue regeneration that may occur during and after the inflammatory response [2]. The pathological fibrotic process of remodeling frequently leads to organ dysfunction and can be associated with high morbidity and mortality.

During this process, a multitude of cell types intervene at different levels, such as epithelial, endothelial, and inflammatory cells, which recruit mesenchymal cells such as fibroblasts and myofibroblasts, leading to fibrosis. This process can be triggered by multiple and different stimuli, like, trauma, wound, infection, metabolic disorders, autoimmunity, inflammation, chronic kidney disease-mineral and bone disorders (CKD-MBD), and others, which converge in molecular signals responsible for initiating and driving fibrosis.

Fibrosis can play a defensive role in some infectious conditions encapsulating the pathogens [3]. It can also play a protective role in cases of important tissue destruction such as in renal infarctions, pyelonephritis, and myocardial infarction. In the latter, the formed fibrotic scar helps to reduce the risk of cardiac rupture [4]. In these circumstances, at least hypothetically, to block the fibrotic repairing process may have undesirable effects [2]. Normal wound healing and fibrosis have many common aspects, in both cases, an injury or a pathogenic factor triggers the cascade of reparation in the affected tissues with the aim to restore the tissue organ integrity [1]. Thus, in certain circumstances, there are no clear limits and definitions of what should be considered physiological and pathological fibrosis.

In normal wound healing, myofibroblasts undergo apoptosis, but the reparative response ends when the damaged tissue has been repaired. By contrast, in pathological fibrosis, tissue remodeling and the myofibroblasts activation and accumulation remain and it becomes an uncontrolled process [5]. Thus, fibrosis can be considered a disproportionately high and prolonged wound healing response. In fact, not only the pathological activation of tissue repair can drive fibrosis, impaired termination of tissue repair can also play a role [1]. An important concept to keep in mind to understand the importance and the impact of fibrosis is the fact that at a certain stage, not well defined, fibrosis progressively becomes irreversible and self-maintained [6,7].

The reparative cascade involves a rapid inflammatory response which leads to leukocyte infiltration and the activation of several molecular pathways which will be discussed later, promoting the activation and accumulation of myofibroblasts [8].

A few years ago, the concept of “core signaling pathways” was introduced to better understand the common and distinct molecular mechanisms of fibrosis in different tissues and fibrotic diseases [9]. The “core signaling pathway” is defined as the “essential pathways to convert an initial stimulus in a pathological fibrosis”. In addition, the concept of “regulatory pathway” was defined as those factors that can “influence the core pathway” but cannot directly convert an initial stimulus in a pathological fibrosis”. In this review, from now onwards, we will refer to the pathological fibrosis simply as “fibrosis” to differentiate it from normal wound healing fibrosis.

Fibrosis in CKD plays a key role at different levels. It has been estimated that CKD affects at least around 10% of the worldwide population and this percentage increases with the ageing [10,11]. In CKD, hypertension, cardiovascular dysfunction, inflammation, abnormal vitamin D metabolism, hyperparathyroidism, hyperphosphatemia, and high serum levels of fibroblast growth factor 23 (FGF23) have been blamed for the extremely high morbidity and mortality of these patients, particularly in CKD stages 3 to 5. Fibrosis in CKD is a progressive process that deteriorates not only the kidney but the heart, causing severe myocardial dysfunction, regardless of cause.

In fact, cardiovascular disease is the main cause of death in patients with CKD [12] and the left ventricular hypertrophy (LVH) with increased fibrosis and cardiac dysfunction are extremely frequent findings [13]. Cardiomyocytes and fibroblasts are involved in the remodeling process which leads to increased myocardial fibrosis in CKD. Cardiomyocytes increase their size, undergo apoptosis or necrosis, and they are replaced by fibroblasts, as a consequence, the collagen synthesis is increased leading to fibrosis [14,15,16].

**Figure 1:**
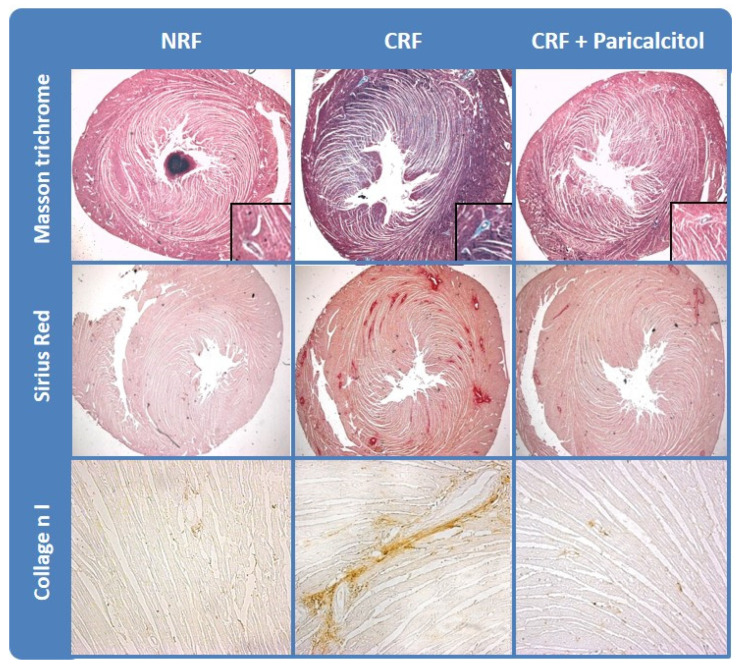
Vitamin D receptor activators (VDRAs) reduced cardiac fibrosis in experimental chronic kidney disease (CKD). Cardiac fibrosis in rats with normal renal function (NRF) and chronic renal failure (CRF) analyzed by collagen deposition (Masson Trichrome and Sirius Red stainings) and Collagen I immunohistrochemistry. Modified from Panizo et al. Nephrol Dial Transplant 2013 28(11):2735-44 with permission of Oxford University Press.

Secondary to this remodeling process, there is an increase in the heart size with elevated proportion of fibrotic tissue at the expenses of a proportional reduction in cardiac muscular functional tissue [14] (Figure 1). These changes, together with the increased stiffness of the main arteries, mainly due to the parallel increase in vascular calcification [17], are the more important factors responsible of the high prevalence of cardiovascular disease in CKD patients.

**Figure 2:**
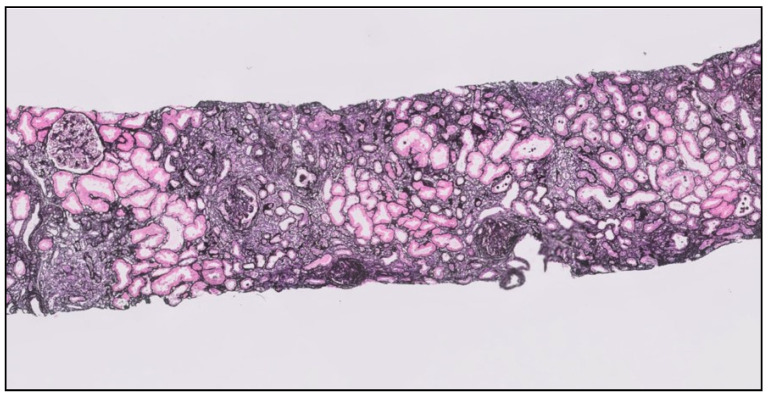
Kidney biopsy showing an advanced stage of tubule interstitial and glomerular fibrosis (T2) in a patient with IgA nephropathy (Jones silver stain ×400).

Besides the cardiovascular impact described above standing for kidney fibrosis, it progressively reduces the renal function, replacing within the renal parenchyma, parts of the functional kidney by scars. The fibrotic matrix deposition disrupts the kidney architecture and reduces the blood supply, impairing the kidney function and ultimately causing irreversible kidney failure [18]. This process, of excessive connective tissue accumulation mainly produced by the myofibroblasts, combines features of smooth muscle cells and fibroblasts. It affects all compartments of the kidney, starting in the tubulointerstitial area and expanding into the vasculature and glomeruli, leading to arteriosclerosis and glomerulosclerosis, representing the common pathological pathway followed for almost all chronic nephropathies [18,19] (Figure 2).

Several systems, molecules, and responses involved in the pathogenesis of fibrosis in CKD—both, core or regulatory signaling pathways—will be discussed in this review, particularly inflammation, renin-angiotensin system (RAS), parathyroid hormone (PTH), FGF23/klotho, microRNAs (miRs), and the vitamin D hormonal system.

## 2. Inflammation and CKD Fibrosis

The early stages of the fibrotic process are characterized by complex inflammatory events involving the innate and adaptive immunity [1]. The early inflammatory phase of the wound healing response depends on both M1 macrophages and M2 reparative macrophages [20,21]. In contrast to these acute inflammatory reactions, in the early phases of the organ injury, fibrosis typically results from chronic inflammation as a consequence of an immune response that persists for several months, in which inflammation, tissue remodeling, and repair processes occur simultaneously [8]. Thus, chronic inflammation, characteristic of CKD, is often the trigger for the fibrosis process. The inflammatory process is transmitted through epithelial and endothelial cells, which give rise to inflammatory mediators including cytokines and chemokines among others [22,23], and leads to the recruitment of inflammatory cells: lymphocytes, polymorphonuclear leukocytes, eosinophils, basophils, mast cells, and macrophages. These inflammatory cells release transforming growth factor beta 1 (TGF-ß1), a potent fibrogenic factor that induces the activation of fibroblasts, increasing the synthesis of extracellular matrix (ECM) proteins [24,25,26].

It is well recognized that renal inflammation is the fuel for the initiation of renal fibrosis. In acute and chronic kidney injury, the release of cytokines, infiltration of inflammatory cells, and subsequent epithelial to mesenchymal transition (EMT) lead to renal fibrosis and failure [27,28]. Tubular repair mechanisms involve epithelial growth factor receptor (EGFR) activation. Although its acute activation is beneficial in the early stages of kidney injury, its chronic activation leads to renal fibrosis [29,30,31,32,33,34]. This activation increases the expression of TGF-ß1, which stimulates interstitial myofibroblast proliferation, inducing the secretion of collagen and other ECM proteins, leading to interstitial fibrosis and functional failure of nephrons.

Another important factor involved in inflammation and fibrosis is a member of a disintegrin and metalloproteinase (ADAM) family namedADAM17 o TACE (tumor necrosis factor-alfa converting enzyme), which releases proforms of EGFR, and tumor necrosis factor-alfa (TNF-α) to produce active soluble ligands, thus further exacerbating the problem [35]. Acute or chronic kidney damage causes a sustained elevation not only of the levels of ADAM17, but also of its substrates TNF-α and amphiregulin with its corresponding receptors. As a consequence, EGFR, the major ligand for amphiregulin, is persistently activated, increasing the synthesis and release of pro-inflammatory and profibrotic factors. The inhibition of ADAM17 in the proximal tubule protects against these effects. In vitro, in proximal tubule cells, amphiregulin has unique profibrotic actions that are increased by TNF-α-induced cleavage of amphiregulin. In patients with AKI and CKD, soluble amphiregulin is highly upregulated in urine, and both the expression of ADAM17 and amphiregulin show a strong positive correlation with the fibrosis markers in kidney biopsies [19].

Although myofibroblasts are a major source of excessive ECM proteins production in the cardiac fibrosis, macrophages also contribute to the excess of these proteins and remodeling. After cardiac damage, the release and increase of IL-10, insulin growth factor 1 (IGF1), TGF-β1, and Galectin-3 by modified macrophages induces ECM remodeling [36,37]. On the other hand, cardiomyocytes and cardiac fibroblasts secrete proinflammatory cytokines, inducing an increase of cardiac fibrosis by myofibroblasts [38,39,40].

## 3. The Renin-Angiotensin System and CKD Fibrosis

The renin-angiotensin system (RAS) is recognized for its important function in the control of extracellular fluid volume and arterial pressure [41]. Chronic RAS activation has a key role in the pathogenesis of renal and cardiovascular disorders like hypertension, heart failure, and fibrosis, among others [42]. Even though the RAS was firstly recognized to play a key role in the circulatory system, this concept has been expanded to other tissues due to the local existence of many of its components in different tissues like heart, kidney, liver, and lung [43]. Local RAS is involved in the injury, inflammatory, and fibrogenic diseases of many organs including the kidney [44] and heart [45] by independent mechanisms of the circulating RAS. In addition, angiotensin II (Ang-II) acts through specific receptors on kidney, cardiovascular system, and other tissues [46,47,48].

RAS components can be classified in two individual pathways, the classical and the alternative pathway. The classical RAS pathway activation started with the synthesis of renin and angiotensin. Renin, an aspartyl protease, is secreted by the juxtaglomerular cells in the kidney and it is also a component that limits the rate of reaction in the RAS [49]. The main function of renin is the production of angiotensin I (Ang-I), a fragment of 10 amino acid peptide of angiotensinogen cleaved by renal renin. Ang-I is transformed by the angiotensin-converting enzyme (ACE) into the octapeptide Ang-II that regulates blood pressure and is a key player in hypertension. Ang-II is the active component of RAS and exerts its actions through interactions with two distinct G-protein-coupled receptors Ang-II type 1 (AT1R) and type 2 (AT2R) receptor [50] in different tissues such as the kidney and heart [51]. Furthermore, Ang-II is the most active component of the RAS, its known effect in the synthesis of collagen and fibronectin stimulates the Smad signaling pathways by TGF-ß-independent mechanism [52]. Ang-II also activates TGF-ß1pathways [53], indicating that RAS plays an important role in tissue fibrosis [45,54], thus RAS can also be considered another “core” signaling pathway.

On the other hand, in the alternative RAS pathway, the ACE2 and its product Ang (1-7) binds to the seven-transmembrane G-protein-coupled receptor (Mas receptor). The corresponding ACE2/Ang (1-7)/Mas receptor axis had a protective function in the fibrogenesis and inflammation in many organs, antagonizing the effects of the classical RAS pathway [55]. ACE and ACE2 zinc metalloproteases, with a homology of 42% in the catalytic domain, have antagonist functions. In a model of cardiac fibrosis in rats, it has been shown that the induction of cardiac fibrosis by Ang-II was prevented by the administration of Ang (1-7). [56]. Furthermore, in hypertensive rats, the administration of Ang (1-7) decreased fibrosis [57] and the overexpression of ACE2 in mice reversed cardiac hypertrophy and fibrosis [58], meanwhile ACE2 deficiency resulted in progression of cardiac and renal fibrosis [59,60].

Besides, RAS activation induces ADAM17 and promotes inflammation and fibrosis [61,62,63]. Some studies have reported the existence of pro-inflammatory components of the RAS in the kidney and heart [64,65], where ADAM17 is expressed. In the kidney, when Ang-II binds to its receptor AT1R, RAS activation induces ADAM17 translocation and migration to the cell membrane. Once the Ang-II triggers the process of kidney damage, the release of pro-fibrotic and pro-inflammatory cytokines causes systemic inflammation, aggravating kidney damage [47,66].

The implications of RAS in the control of extracellular fluid volume, hypertension, inflammation, and renal and cardiac fibrosis have led to the development of effective therapies [56,67]. Multiple drugs have been studied to interfere with RAS at different levels, such as renin inhibitors, ACE inhibitors, Ang-II receptor blockers (ARBs), and mineral receptor antagonists, all these compounds can attenuate or prevent cardiac and/or renal fibrosis.

## 4. Age, Sex, PTH, Phosphate, FGF23, Klotho, and Fibrosis

Several key parameters in the evolution of CKD, such as age, sex, PTH, phosphate, FGF23, and Klotho, among others, are also involved in the development of renal and cardiac fibrosis.

Progressive fibrosis is an indication of aging in various organs such as kidney and heart [68]. In normal individuals, the number of nephrons is reduced with the age and fibroblast proliferates. Activated renal fibroblasts deposit excess extracellular matrix proteins, and kidney function declines [69]. This is the beginning of the deterioration of kidney structure and function that can lead to the final stages of CKD and even the need for renal replacement therapy such as dialysis or transplantation.

Cardiac aging is associated with significant alterations in cardiac structure and function. In the senescent heart, there is hypertrophy of cardiomyocytes, transition of fibroblasts to myofibroblasts, and accumulation of extracellular matrix proteins in the interstitium. These alterations lead to perivascular, endomysial, and perimysial fibrosis [68]. Levels of myocardial fibrosis are more severe in CKD patients, especially on dialysis patients, and partially regressed in patients with renal transplantation [70].

In the heart of CKD patients, the gender can influence the pathological cardiac remodeling [71], in male, harmful effects due to the hormone’s role in the myocardium have been shown because male hormones can increase RAS activation, worsening fibrosis. By contrast, estrogens seems to be protectors attenuating adverse cardiac remodeling [71]. This fact is also observed in the kidney, where female hormones can have a renoprotective effect [72].

In CKD, secondary hyperparathyroidism has been associated with fibrosis [73,74]. PTH can increase cardiac fibrosis, acting through direct or indirect mechanisms: Directly, stimulating collagen deposition [73], promoting the endothelial-mesenchymal transition [74], stimulating the aldosterone release [75], and increasing the calcium channel TRPV6 (transient receptor potential channels, of the vanilloid subtype 6) and thus the cardiac intracellular calcium content. Indirectly, by the stimulation of the growth of the cardiomyocytes inducting LVH, which in turns increase the proportion of fibroblasts leading to cardiac fibrosis [76]. Part of these mechanisms are shared with renal fibrosis [74], where in addition, PTH enhances connective tissue growth factor (CTGF) expression in proximal tubular cells [77].

Serum phosphate and FGF23, which are elevated in the advances stages of CKD [78], are able also to induce cardiac hypertrophy [79,80], and they play an important role in myocardial fibrosis through the stimulation of β-catenin and the TGF-β pathway [81]. In the kidney, FGF23 induces fibrotic signaling cascades via activation of TGF-β [82,83], by Klotho independent mechanism [83].

However, Klotho also regulates TGF-β1 signaling, leading to kidney fibrosis. In fact, Klotho downregulation allows the development of a full profibrotic response, and studies in humans suggest the use of recombinant Klotho, Klotho-derived peptides, or the control of factors that decrease Klotho expression may be useful in the treatment of kidney injury [84].

As the changes in serum PTH, phosphate, FGF23, and Klotho are tightly interrelated, it is difficult to dissect their individual participations, though PTH, the most studied factor, plays a key role in the fibrotic process.

## 5. MicroRNAs and CKD Fibrosis

MicroRNAs (miRs) are small 21–25 nucleotide single-stranded non-coding RNA molecules, which negatively regulate the expression of their target genes. MiRs have a key role in the physiological regulation of distinct processes in mammals, such as the control of heart and kidney development, structure, and function; but they are also involved in pathological processes [16,85,86].

Recent studies suggest that miRs are important in the regulation of the normal kidney function, where they are involved in the regulation of electrolytes, acid-base and fluids balance, and the maintenance of blood pressure [85,87,88]. In addition, they participate in the regulation of normal heart function, modulating the coupling between excitation and contraction [89]. Besides all these positive actions, several studies have shown that miRs are involved in pathophysiological processes such as fibrosis, where they can act as promoters or inhibitors of fibrosis.

Some miRs are regulated by the TGF-ß1 “core signaling pathway”, acting as downstream factors mediating a different pro-fibrotic action, which contributes to the excessive deposition of collagen and tissue fibrosis and they can also facilitate the process of EMT [90,91,92,93,94]. There is a large list of miRs whose overexpression has shown to be able to induce renal and cardiac fibrosis: miR-21 [91,95,96,97], miR-34 [98,99,100] miR-132 [101,102], miR-192 [103], miR-199 [104,105], miR-214 [106], and miR-433 [107]; meanwhile, their downregulation has shown to decrease renal and cardiac fibrosis.

On the other hand, other miRs act as antifibrotic factors, protecting the kidney and the heart, among them, Let-7 [108,109,110], miR-29 [111,112], miR-30 [113,114,115], miR-200 [116], and miR-221/222 [117] families have shown to act as fibrosis inhibitors. The miR-29 family is one of the most studied in fibrosis, they can act downregulating some important targets genes encoding collagen types I and IV and MMP2, among others [111,112]. In fact, the downregulation of miR-29 by TGF-ß1/Smad3 is an important mechanism to induce fibrosis [92], by contrast, the maintenance of the miR29 levels prevents the increase in fibrosis [15]. MiR-30 and miR-133 have been also mentioned as antifibrotic factors in the heart; where they have shown that in the course of LVH, their decrease favors the pro-fibrotic signaling, in part through increases in CTGF levels [114].

MiRs are potential candidates to have a future role in the diagnosis of renal and cardiac abnormalities, due to the fact that they are easily detected in serum by simple and inexpensive techniques with the advantage of their specificity. The latter is particularly important since cardiac and renal fibrosis are difficult to diagnose with non-invasive techniques [118]. A recent study comparing different degrees of renal fibrosis identified a unique miRs profile probably associated with kidney fibrosis [119]. In fact, several miRs have been suggested to be useful for the diagnoses and prognoses of renal fibrosis such as miR-21 and miR-29c [120,121]; and for cardiac fibrosis, such as miR-21, miR-29, miR-30, and miR-133 [15,122,123]. It is interesting to note, that in several cases, the changes in the miR levels in the tissue are opposite to their serum levels; this paradox might be explained by the release of the miRs from the tissue to the circulation, though this observation needs more investigation to be ratified [15,124,125].

MiRs could also allow in the future to design new effective therapeutic strategies to prevent or treat cardiac or renal fibrosis. Nowadays, their use as therapeutic agents is still experimental, as example, the administration of miR-29 in rats may decrease fibrosis in the kidney [92] and heart [126] and in other organs. Other drugs currently used in CKD, such as vitamin D receptor activators (VDRAs), can directly regulate the transcription of miRs, reducing cardiac fibrosis, in part through the maintenance of cardiac miR-29b and miR-30c expression [15]. In summary, the progress in miRs research has been important in recent years though there is still time to go for their translation in clinical practice.

## 6. Diabetes and CKD Fibrosis

Diabetes is one of the primary noncommunicable diseases that affects 463 million people worldwide and this number is estimated to rise to 700 million in 2045 [127]. Diabetes is an important cause of morbidity and premature mortality associated with kidney and cardiovascular disease [128,129] with bidirectional effects. The disorders of the kidney impact the heart and vice versa, in this dual way morbid relationship, fibrosis plays a key role.

In diabetes, the underlying signaling pathways involved in fibrosis are highly complex, with a wide range of functional drivers [130]. Among them, the accumulation of advanced glycation end products (AGES) in both intracellular and extracellular space plays an important role in renal and cardiac fibrosis through several different mechanisms [131,132]. Under hyperglycemic conditions, the reducing sugars react non-enzymatically with amino groups in proteins, lipids, and nucleic acids through a series of intermediate reactions forming Schiff bases and Amadori products that, in the late stage, results in irreversible compounds called AGEs [133]. Another source of AGEs is the food, a recently meta-analysis showed that a diet with high AGE content would be linked to the development of cardio-metabolic risk factors, while a low AGE intake might be beneficial in patients with metabolic disorders such as diabetes, as it reduces the insulin resistance and the cholesterol levels [134]. These AGEs may exert their pathological action mainly through two ways:

(A) Formation of covalent cross-linking with stable and long half-lived proteins in the extracellular matrix, such as collagen, these changes increase heart stiffness, leading to reduced cardiac compliance, mediating diastolic dysfunction [135,136,137]. In the kidney, non-enzymatic glycations of collagen and laminin lead to increase vascular permeability to albumin [138], basement membrane thickening, and mesangial expansion [139], which are hallmarks of diabetic nephropathy. The inhibition of the above-mentioned process can reduce the negative impact of the AGEs in the heart and the kidney [137,140].

(B) Interaction of the AGEs with membrane receptors, mainly RAGE (multi-ligand receptor for AGEs), although others receptors have been identified [141,142]. This interaction is linked to reactive oxygen species (ROS) generation, oxidative stress induction, and fibrosis [131]. Moreover, overexpression of RAGE has been associated to glomerulosclerosis and renal insufficiency [143]. In fact, the gene deletion or pharmacological inhibition of RAGE can effectively prevent the development of the increased left ventricular diastolic chamber stiffness and also blocked the reduction of cardiac systolic function, even in the same hyperglycemic conditions [144]. In addition, the inhibition of RAGE can also reduce the early dysfunction and glomerular structural changes in the kidneys [145,146].

Related to this matter, a novel term has emerged, ”Diabesity”, which combines obesity with diabetes [147,148]. In fact, obesity is a risk factor for diabetes, for each kilogram rise in body weight, the risk of diabetes increases by 4.5% [147]. Metabolic dysfunction, oxidative stress, fasting hyperglycemia, insulin resistance, and dyslipidemias are highly prevalent in obese subjects; they could be implicated in hypertrophic and fibrotic cardiac remodeling [148,149] and also in RAS activation, pressure, and volume overload and inflammation [149]. The increase of pressure in these patients is also associated with glomerular hyperfiltration which induces other mechanisms of renal fibrosis [150], such us tubule-interstitial inflammation, hypoxia, and podocyte hypertrophy [151].

Experimentally, in vitro studies in cardiac fibroblast indicate that AGEs activate several signaling pathways such as p38Map-kinase, extracellular signal-regulated kinase (ERK), and c-Jun N-terminal kinase (JNK), the expression of c-Jun, ATF2, and nuclear factor kB (NF-kB) transcription, as well as changes in matrix metalloproteinases (MMP) expression [152]. Fibroblast isolated from hearts of diabetic mouse showed an increase of AGE levels and RAGE expression as well as an increment of expression and secretion of type I collagen, profibrotic stimulators as TGF-ß, and plasminogen activator inhibitor (PAI-1) compared with non-diabetics [136]. In the kidney, AGE/RAGE activation increased the expression of insulin-like growth factor I and II, platelet-derived growth factor, and TGF-ß in mesangial cells, which in turns mediate the production of collagen, laminin, and fibronectin [145].

Studies in diabetic animal models have shown an increase in collagen levels in cardiac fibroblast of diabetic mice compared with non-diabetic mice with significant up-regulation of profibrotic stimulators such as TGF-ß and PAI-1 [136]. Other authors have proposed a possible crosstalk between RAS and AGE/RAGE pathways in the activation of cardiac fibroblast in diabetes through AGE/RAGE interactions that stimulate local Ang-II production via transduction by AT1R, a process which can be inhibited by losartan [153].

The RAS system has been also mentioned to be involved in the pathological progression of the diabetic renal fibrosis through up-regulation of fibronectin and TGF-ß [154]. In kidney, the upregulation of profibrotic factors could be also mediated by alterations in the protein degradation mechanisms as it is the case of the ubiquitin-proteasome pathway. In fact, the AGE/RAGE activation in glomerular mesangial cells increases the ubiquitination and then the proteasome-mediated degradation of the silent information regulator 2-related protein 1 (Sirt1), which is involved in the cell protection from oxidative stress and in the inhibition of profibrotic markers expression [145]. In summary, in diabetes, the AGE/RAGE axis is another important factor implicated in cardiac and kidney fibrosis.

## 7. Vitamin D and CKD Fibrosis

The endocrine system of vitamin D plays an essential role in calcium homeostasis and bone metabolism. However, the impact of its actions also reaches several other tissues and systems where the “non-classical effects” of vitamin D have great importance. Vitamin D is synthesized in the skin as cholecalciferol (vitamin D_3_) or ingested in the diet as cholecalciferol or ergocalciferol (vitamin D_2_). Both are inactive forms of vitamin D that are transported to the liver, where they are metabolized to 25-hydroxyvitamin D (the main storage form of vitamin D). The kidney is the place where the conversion of 25-hydroxyvitamin D to 1,25-dihydroxyvitamin D or calcitriol, the most active natural metabolite of the vitamin D hormonal system takes place. To have “sufficient 25-hydroxyvitamin D” is necessary for the production of renal and extrarenal calcitriol.

**Figure 3:**
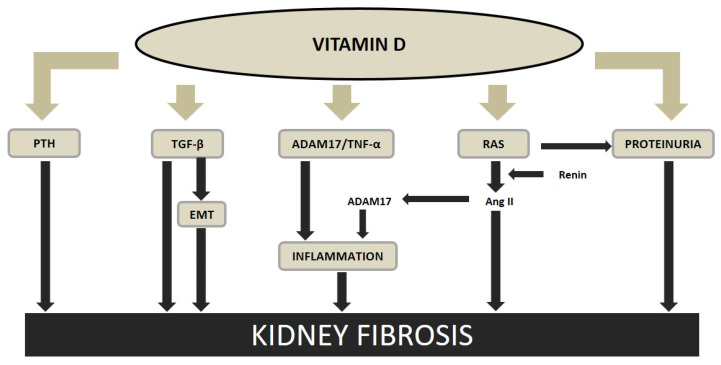
Vitamin D and kidney fibrosis. Thick lines indicate direct interaction, thin lines indicate mediated interaction. PTH, parathyroid hormone; TGF-ß, transforming growth factor beta; EMT, epithelial to mesenchymal transition; ADAM17, tumor necrosis factor-alfa converting enzyme; TNF-α, tumor necrosis factor-alfa; RAS, renin-angiotensin system; Ang II, angiotensin II. Modified from Martinez-Arias et al. Nephrol Dial Transplant 2020 (in press), with permission of Oxford University Press.

Vitamin deficiency and insufficiency have been associated with a higher morbidity and mortality in normal population and in CKD patients. The renoprotective effect of the vitamin D receptor activators (VDRAs) on the development and progression of renal fibrosis could be mainly due to; (a) the role of vitamin D inhibiting the inflammatory process, (b) the vitamin D RAS downregulation, (c) the prevention of EMT, and (d) the reduction of PTH, among others (Figure 3).

Treatment with VDRAs has shown to have a renoprotective effect in part attributed to their anti-inflammatory actions [155]. Several experimental studies have reported that the administration of VDRAs reduces the presence of inflammatory cells in the kidney [155,156,157]. In fact, a recent experimental study, has shown that paricalcitol, one of the most studied and used VDRAs, was able to prevent the increase in renal TNF-α and the inflammatory infiltration induced by the uremia [158] (Figure 3).

The activation of the vitamin D receptor (VDR) by paricalcitol sequesters the transcription factor NF-κB, preventing the transcription of RANTES (regulated on activation, normal T cell expressed and secreted), a cytokine expressed and secreted by activated T lymphocytes [157]. Other antifibrotic actions of VDRAs in the kidney are the inhibition of: TGF-β1, collagen I, thrombospondein 1, and ADAM17; together with the reduction of the leukocyte infiltration [158], antiproliferative effect on mesangial cells; reduction of podocyte hypertrophy and maintenance of its structure; and induction of the expression and secretion of the endogenous antifibrotic factor hepatocyte growth factor (HGF) [150] (Figure 3).

Besides all the mentioned beneficial actions, the VDRAs block the RAS, preventing further kidney damage and the progression of renal fibrosis [158,159] (Figure 3). Vitamin D deficiency activates the intrarenal RAS, which is an important mediator of kidney damage, increasing angiotensin II [160,161,162,163,164]. Thus, vitamin D a negative regulator of the RAS, protects the kidney, reducing the renin gene expression directly inhibiting the transcription of the renin gene in the RAS [165,166]. It has been shown that VDR knockout mice and 1-α-hydroxylase deficient mice feature elevated blood pressure [165,167]. In addition, in experimental CKD, paricalcitol was able to suppress the activation of the renin-angiotensin, reducing glomerular and tubulointerstitial damage and proteinuria.

In addition to the anti-inflammatory and anti RAS effects, it has also been described, that paricalcitol prevents interstitial fibrosis, reducing the changes in EMT [158,168,169]. In the obstructed kidney, paricalcitol prevented the renal fibrosis and suppressed the production of matrix proteins such as collagens I, III, and TGF-ß1 [158,168], and in a CKD induced by puromycin, calcitriol prevented the interstitial fibrosis [170]. Moreover, paricalcitol preserved E-cadherin and abolished TGF-β–mediated E-cadherin suppression in tubular epithelial cells [158,168] (Figure 3).

The combination of the down-regulation of all mentioned factors; renal inflammation, RAS activation, and epithelial/mesenchymal transition by VDRAs, together with its well-known affects reducing PTH and overexpressing Klotho, may explain a great part of the protective effects of the VDRAs on renal and cardiac fibrosis.

In the heart, the beneficial effects of VDRAs reducing myocardial fibrosis and consequently recovering the fibrotic–muscular tissue ratio have been mentioned as one of the VDRAs important actions. In fact, an experimental study has shown that paricalcitol attenuated the cardiac hypertrophy and fibrosis [14], by reducing collagen I, TGF-β1 and increasing MMP1, an interstitial collagenase that degrades type I, II, and III structural collagens, favoring the reduction of collagen [14,171].

These experimental data are partly in agreement with the findings described in the PRIMO (Paricalcitol Capsule Benefits in Renal Failure Induced Cardiac Morbidity) trial performed in CKD stages 3–4 patients [172,173,174], in which despite paricalcitol treatment, failed to reduce left ventricular mass, it showed positive effects on some functional cardiac markers, improving left atrial volume and the diastolic function and reducing cardiovascular hospitalizations.

Even though more research is needed in this area, part of the antifibrotic actions of the VDRAs could be also mediated by their positive effect of maintaining miR-29b and miR-30c levels, already mentioned, which have been associated with prevention or attenuation of the fibrosis [14,15].

The VDRAs beneficial effects have been also described in the diabetic nephropathy due to the links between VDRAs and the glucose metabolism through the upregulation of the glucose transporter 4 (GLUT4) [175] and insulin secretion [176]. Moreover, a recent meta-analysis of randomized controlled trials found that supplementation with VDRAs to type-2 diabetic patients may reduce inflammation [177], down-regulating RAGE gene expression in peripheral blood mononuclear cells and decreasing serum AGES and TNF-α levels [178]. In addition to the protective anti-inflammatory effect of VDRAs on the diabetic nephropathy, several clinical and experimental studies have attributed to the VDRAs a role in the control of the progression of diabetic nephropathy, mainly through their combined effects on the RAS, inflammation, and suppression of the renal tubular EMT. [179]. The protective role of VDRAs in the development of diabetic nephropathy seems to be well accepted, but their potential beneficial role in diabetic cardiomyopathy still needs to be consolidated [180].

In summary, the vitamin D hormonal system is implicated in several steps of the fibrotic process. Due to the ubiquity of the VDR, it is well known that its activation in several tissues is necessary for an efficient up and down regulation of several proteins. Thus, the vitamin D deficiency deregulates important pathways that control the “classical and non-classical” effects of the vitamin D hormonal system. Thus, due to the ubiquity of the VDR present at least in 30 tissues, it is reasonable to consider that the use of VDRAs in several circumstances could be associated to several benefits, including the reduction of renal and cardiac fibrosis.

## 8. The Future in the Diagnosis of CKD Fibrosis

Kidney fibrosis has proven to be one of the most important predictors of evolution to CKD. In such matter, the percentage of kidney fibrosis is estimated by the pathologist through eye recognition and experience and reported in order to subclassify patients in many classifications of different nephropathies such as the Oxford Classification of IgA Nephropathy, Lupus Nephritis, the Banff scoring system in kidney transplantation and many others [181,182,183]. In most of these classifications there are cutoffs based on the percentage of kidney fibrosis that subclassify patients in 3 or 4 different groups.

In the last decade, the introduction of digital pathology has opened a window to computational image analysis and the development of specific algorithms by artificial intelligence which will progressively have diagnostic and prognostic applications.

The application of image analysis through machine learning and artificial neural networks in digital histological images has demonstrated high reproducibility and precision discriminating in very low percentages, which in many instances, is better than the human eye of experienced pathologists. In the following years, the integration of these findings in everyday diagnosis may enable a better characterization of patients in order to predict their outcome and more personalized therapeutic approaches that will lead to precision medicine.

## 9. Translation of the Present Knowledge to the Management of CKD-Related Fibrosis

Not only should the diagnosis of CKD-related fibrosis be more precise in the near future, the important volume of present basic and clinical information on this subject, gathered through several years, should be translated, as much as possible, into the practice, impacting the management of fibrosis in CKD patients. As we have described in the different parts of this review, the CKD-related fibrosis is multifactorial, some of the factors driving to fibrosis such as gender, age, and other human individual risk factors, cannot be modified, but it is possible to act on a non-negligible number of important factors related to fibrosis in the clinical practice (Table 1).

Nowadays, there are several scientifically supported strategies that should be considered in order to prevent and/or reduce the progression of renal fibrosis, including the mitigation of the impact of ageing in kidney and cardiac fibrosis. We have described in detail in the text, from a pathogenic and pathophysiological point of view, the main mechanisms implicated in this process and the strategies to reduce them. In the table, aiming to translate them to practice, we have summarized the main aspects of the clinical management in which we consider there is room for improvement and we can take actions to better manage the CKD-related fibrosis.

## Figures and Tables

**Table 1:** Possible influence of clinical management in the reduction of factors related to Chronic Kidney Disease (CKD)-related fibrosis. RAS, renin-angiotensin system; CKD-MBD, chronic kidney disease-mineral and bone disorders; PTH, parathyroid hormone; P, phosphate; Ca, calcium; FGF23, fibroblast growth factor 23; AGES, advanced glycation end products.

Clinical Management (Actions)	Main Possible Benefits
Reduction of the RAS activation	Better control of extracellular volume Reduction of blood pressure/inflammation Prevention/Reduction of renal/cardiac fibrosis Association with better outcomes (morbidity/mortality)
Adequate control of the CKD-MBD biochemical parameters (PTH, P, Ca)	Reduction of FGF23 Prevention/Reduction of renal/cardiac fibrosis Association with better outcomes (morbidity/mortality)
Adequate control of the glycemia and metabolic parameters of diabetes	Reduction of AGES Reduction of oxidative stress Improvement of lipid profile Improvement of insulin resistance Improvement of cardiac remodelling Reduction of glomerular hyperfiltration Prevention/Reduction of renal/cardiac fibrosis Association with better outcomes (morbidity/mortality)
Normalization of the vitamin D hormonal system (Normalization of 25(OH)D_3_ serum levels)	Improvement of CKD-MBD parameters Reduction of renal and vascular inflammation Downregulation of RAS and upregulation of Klotho Reduction of glomerulotubular damage and proteinuria Improvement of glucose metabolism Prevention/reduction of renal/cardiac fibrosis Association with better outcomes (morbidity/mortality)

## Data Availability

Not applicable.
